# Predictors of Lethality, Remodeling, and Aorta-Related Events in Different Types of Proximal Aortic Dissection Surgery

**DOI:** 10.17691/stm2023.15.1.05

**Published:** 2023-01-28

**Authors:** D.A. Sirota, М.О. Zhulkov, D.S. Khvan, T. Caus, B.N. Kozlov, V.L. Lukinov, М.М. Lyashenko, A.G. Makaev, A.V. Protopopov, Kh.A. Agaeva, A.V. Fomichev, S.A. Мagbulova, A.D. Limansky, A.М. Chernyavsky

**Affiliations:** Head of the Research Department of Surgery on Aorta, Coronary and Peripheral Arteries, Institute of Blood Circulation Pathology; Meshalkin National Medical Research Center of the Ministry of Health of the Russian Federation, 15 Rechkunovskaya St., Novosibirsk, 630055, Russia; Cardiovascular Surgeon, Department of Aorta and Coronary Artery Surgery; Meshalkin National Medical Research Center of the Ministry of Health of the Russian Federation, 15 Rechkunovskaya St., Novosibirsk, 630055, Russia;; Researcher, Research Department of Surgery on Aorta, Coronary and Peripheral Arteries, Institute of Blood Circulation Pathology; Meshalkin National Medical Research Center of the Ministry of Health of the Russian Federation, 15 Rechkunovskaya St., Novosibirsk, 630055, Russia; Cardiovascular Surgeon, Department of Aorta and Coronary Artery Surgery; Meshalkin National Medical Research Center of the Ministry of Health of the Russian Federation, 15 Rechkunovskaya St., Novosibirsk, 630055, Russia;; Senior Researcher, Research Department of Surgery on Aorta, Coronary and Peripheral Arteries, Institute of Blood Circulation Pathology; Meshalkin National Medical Research Center of the Ministry of Health of the Russian Federation, 15 Rechkunovskaya St., Novosibirsk, 630055, Russia; Cardiovascular Surgeon, Department of Aorta and Coronary Artery Surgery; Meshalkin National Medical Research Center of the Ministry of Health of the Russian Federation, 15 Rechkunovskaya St., Novosibirsk, 630055, Russia;; Cardiovascular Surgeon; University Hospital of Amiens, Avenue René Laënnec, Salouël, Amiens, 80054, France;; Head of the Cardiovascular Surgery Department; Cardiology Research Institute, Tomsk National Research Medical Center of the Russian Academy of Sciences, 111a Kievskaya St., Tomsk, 634012, Russia;; Senior Researcher; Institute of Computational Mathematics and Mathematical Geophysics, Siberian Branch of the Russian Academy of Sciences, 6 Academician Lavrentyeva Prospect, Novosibirsk, 630090, Russia; Head of the Laboratory of Numerical Analysis of Stochastic Differential Equations; Institute of Computational Mathematics and Mathematical Geophysics, Siberian Branch of the Russian Academy of Sciences, 6 Academician Lavrentyeva Prospect, Novosibirsk, 630090, Russia;; Cardiovascular Surgeon; Meshalkin National Medical Research Center of the Ministry of Health of the Russian Federation, 15 Rechkunovskaya St., Novosibirsk, 630055, Russia; Head of the Department of Surgery on Aorta, Coronary and Peripheral Arteries; Meshalkin National Medical Research Center of the Ministry of Health of the Russian Federation, 15 Rechkunovskaya St., Novosibirsk, 630055, Russia;; Resident, Cardiovascular Surgeon; Meshalkin National Medical Research Center of the Ministry of Health of the Russian Federation, 15 Rechkunovskaya St., Novosibirsk, 630055, Russia;; Resident, Cardiovascular Surgeon; Meshalkin National Medical Research Center of the Ministry of Health of the Russian Federation, 15 Rechkunovskaya St., Novosibirsk, 630055, Russia;; Cardiovascular Surgeon, Department of Surgery on Aorta, Coronary and Peripheral Arteries; Meshalkin National Medical Research Center of the Ministry of Health of the Russian Federation, 15 Rechkunovskaya St., Novosibirsk, 630055, Russia;; Senior Researcher, Research Department of Surgery on Aorta, Coronary and Peripheral Arteries, Institute of Blood Circulation Pathology; Meshalkin National Medical Research Center of the Ministry of Health of the Russian Federation, 15 Rechkunovskaya St., Novosibirsk, 630055, Russia; Cardiovascular Surgeon, Department of Surgery on Aorta, Coronary and Peripheral Arteries; Meshalkin National Medical Research Center of the Ministry of Health of the Russian Federation, 15 Rechkunovskaya St., Novosibirsk, 630055, Russia;; Resident, Cardiovascular Surgeon; Novosibirsk State Medical University, 52 Krasny Prospect, Novosibirsk, 630091, Russia;; Student; V. Zelman Institute for Medicine and Psychology, Novosibirsk State University, 1 Pirogova St., Novosibirsk, 630090, Russia; Professor, Correspondent Member of the Russian Academy of Sciences, General Director; Meshalkin National Medical Research Center of the Ministry of Health of the Russian Federation, 15 Rechkunovskaya St., Novosibirsk, 630055, Russia;

**Keywords:** “frozen elephant trunk”, aorta dissection, aortic arch, thoracic aorta, thrombosis, substantial hemorrhage, stents

## Abstract

**The aim of the study** is to analyze predictors of lethality, false lumen thrombosis, enlargement of aortic diameter, and frequency of aorta-related events in the early and remote postoperative periods for various types of proximal aortic dissection surgery using the logistic regression method.

**Materials and Methods:**

A retrospective observational comparison of the results of surgical treatment of 213 patients with the diagnosis of “DeBakey type I aortic dissection” has been carried out. The participants were divided into three groups: group 1 underwent classic aortic arch reconstruction using hemiarch technique or total reconstruction of the aortic arch with a multiple-branch prosthesis (n=121); group 2 was subjected to the hemiarch technique and implantation of bare-metal (uncoated) stents (n=55); in group 3, the “frozen elephant trunk” correction technique was used (n=37). The diagnosis of all patients included into the study was preoperatively confirmed by ultrasound and tomographic examination. Predictors of negative events have been identified by building the models of logistic regressions.

**Results:**

The multivariate model of logistic regression has revealed multiplicative significant predictors of lethality: postoperative neurological complications increased the probability of lethality by 3.39 (1.24–9.18) times and presence of a patent false lumen by 4.17 (1.49–13.68) times.

Among the predictors of aorta-related events, the most important were connective tissue diseases (the probability increased by 6.68 (2.98–15.62) times), presence of partial thrombosis of the false lumen (the probability of event development increased by 2.39 (1.07–5.44) times), and aortic valve repair (the probability aorta-event occurrence increased by 2.84 (1.13–7.17) times).

Hybrid prosthesis implantation appeared to be the most significant predictor of false lumen thrombosis increasing its probability by 4.19 (1.90–9.44) times among aortic repair methods, while a bare-metal stent implantation in contrast reduced the likelihood of false lumen thrombosis by 0.17 (0.03–0.62) times. Eventually, the type of repair had not any significant impact on the aorta-related events and lethality in the long-term period.

## Introduction

Despite many factors aggravating essentially the condition of patients with proximal aortic dissection, the results of surgical treatment of this cohort of patients are gradually improving owing to timely diagnosis and the surgical intervention performed. Nevertheless, the rate of hospital lethality remains within the range of 10– 30% [1-6].

Presently, concurrently with classic (traditional) intervention, i.e. repair of the ascending aorta and the arch using hemiarch or total arch replacement techniques, implantation of supplementary devices such as bare-metal stents or stent grafts into the descending aorta has become technically feasible [7]. However, the effectiveness of these extended interventions on aorta has been insufficiently studied so far.

Having analyzed the results of the standard approach to the aorta reconstruction, Suzuki et al. showed in their study [8] that actuarial freedom from reoperations was 96.9, 83.2, 64.2, and 58.3% after 1, 5, 10, and 12 years, respectively. Regression analysis of Cox proportional hazards allowed the authors to identify the following independent predictors of late repeated operation: young age, malperfusion, proximal fenestration in the ascending aorta, descending aorta diameter, F/T>1 index (ratio of false and true lumen diameter in the region of the descending aorta), and the Marfan syndrome.

Presence of the patent false lumen is one of the main predictors of postoperative aorta remodeling, and therefore the risk of repeated interventions — it is the very postulate that became the basis of the surgical strategy of treating aortic dissection [9]. Despite the desire for maximum readaptation of the dissected aortic walls, the effectiveness of various surgical techniques in achieving this goal differs considerably. Thus, the “elephant trunk” operation, suggested by H.G. Borst in 1983, did not contribute to turning off aneurysm and/or false lumen from the bloodstream [10, 11]. The advent of new prostheses and technologies moved surgeons along the path of greater radicalism in performing primary reconstructions of proximal aortic dissections. However, advantages of these interventions are still unobvious. For example, the effectiveness of Djumbodis Dissection System implantation (a bare-metal stent) has repeatedly been called into question. According to the literature data, stenting of the true lumen with a bare-metal stent results in thrombosis of a false lumen only in 68% of cases in acute and in 18–20% in chronic dissection, which cannot compete with actually 100% thrombosis over 5 years using the technique of “frozen elephant trunk” [7, 12–14].

Although the results of surgical treatment of proximal aortic dissections are gradually improving with time, operation lethality remains steady within 18–25% [5]. Taking into consideration anatomical variability of aortic dissections and a wide choice of surgical correction techniques, the analysis of aorta-related event predictors and the resulting repeated surgical interventions is of great interest [15].

**The aim of the study** is to analyze predictors of lethality, false lumen thrombosis, enlargement of aortic diameter, and frequency of aorta-related events in the early and remote postoperative periods for a variety of proximal aortic dissection repair using the logistic regression method.

## Materials and Methods

A retrospective observational study has been performed. The results of surgical treatment of 213 patients with the diagnosis of “DeBakey type 1 aortic dissection” were compared. In all patients, dissection involved ascending aorta, aortic arch, and descending thoracic aorta. Patients operated on in the clinics of Meshalkin National Medical Research Center (Novosibirsk, Russia), Cardiology Research Institute of Tomsk National Research Medical Center of the Russian Academy of Sciences (Russia), and University Hospital of Amiens (France) from 2001 to 2017 were included in the study.

**Figure 1.**
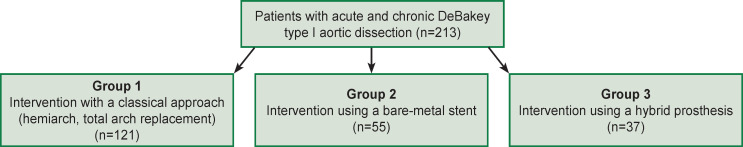
Study design

Participants were divided into three groups: in group 1, standard surgical approaches were applied (hemiarch technique or total reconstruction of the aortic arch (n=121)); group 2 was subjected to the hemiarch technique and implantation of bare-metal stents (n=55); in group 3, the “frozen elephant trunk” technique was used (n=37) (Figure 1).

The diagnosis of all patients included into the study was preoperatively confirmed by ultrasound and tomographic examination. False lumen thrombosis was assessed over the whole lumen length irrespective of the anatomical zone. Absence of false lumen contrast was considered as total thrombosis, no signs of false lumen thrombosing denoted complete patency defined by the contrast-enhanced MSCT examination. Any events related to aorta (ruptures, dissections) as well as interventions on aorta and aortic valve over the entire follow-up period were referred to aorta-related events.

### Statistical data processing

Sampling distribution of continuous indicators was checked for normality of distribution using the Shapiro–Wilk test. The terms of observation appeared abnormal, therefore, comparison was carried out with the help of non-parametric Mann– Whitney U-test with the correction of multiple comparison error using the Benjamini–Hochberg test. To assess the value of difference in the groups, a pseudomedian of value differences and standardized mean difference (SMD) were calculated. The continuous indicators were presented as median, 25^th^ and 75^th^ percentiles (Me [Q1; Q3]), mean and standard deviation (M±SD). Binary indicators were described as the number of events and frequency with the construction of a 95% confidence interval using the Wilson formula (n/%, 95% CI).

Negative event predictors were identified building the models of logistic regressions. By means of single-factor models, separate predictors associated with a target event were determined. Covariate population with the achieved level of significance of p<0.3 in single-factor models was employed to build models of multifactor logistic regression optimally complying with the Akaike information criterion using forward and backward stepwise selection method. All models of the forward and backward step coincided. For the multifactor model of the logistic regression, the best classification threshold in terms of sensitivity/specificity ratio was detected by the ROC analysis methods, the contingency table was built to calculate prognostic indicators: sensitivity, specificity, frequency of method occurrence, actual frequency of occurrences. Goodness of fit for prognostic frequencies of the calibrated model and actual frequencies of negative events was studied by means of the Hosmer– Lemeshow test.

Statistical hypotheses were checked at the critical level of significance of p=0.05, i.e. differences were considered statistically significant at p<0.05.

All statistical calculations were performed in R-Studio program (version 2022.07.2+576 Spotted Wakerobin, USA) using R language (version 4.1.3, Austria).

## Results

The patients were followed up in-person or remotely. A mean period of observation was calculated separately for each group (Table 1).

**Table 1:** Terms of patient follow-up in each group

Groups	Follow-up duration (months)			Groups	Differences between the groups		
	Me [Q1; Q3]	M±SD	Min–max		Pseudomedian (95% CI)	SMD (95% CI)	p* (p correction)
1	35.0 [5.0; 57.0]	34.53±30.15	0–103	1–2	3 (–2–12)	0.21 (–0.11–0.53)	0.235 (0.471)
2	32.0 [5.5; 47.0]	28.47±24.16	0–100	1–3	7 (–2–19)	0.35 (–0.02–0.72)	0.103 (0.310)
3	22.0 [11.0; 36.0]	24.68±21.51	0–97	2–3	4 (–5–13)	0.16 (–0.25–0.58)	0.344 (0.471)

* the Mann–Whitney U-test.

The descriptive statistics of the examined covariates is presented in Table 2.

**Table 2:** Descriptive statistics of the examined indicators

Covariates	Number of data	Statistics	Values
Male gender	213 (100%)	n/%, 95% CI	136/64, 57–70
Body mass (kg)	204 (96%)	Me [Q1; Q3] M±SD Min–max	78 [68; 90] 79.73±17.61 42–146
Body height (cm)	203 (95%)	Me [Q1; Q3] M±SD Min–max	173 [166; 179] 172.71±9.88 147–196
Connective tissue diseases	213 (100%)	n/%, 95% CI	49/23, 18–29
Absence of arterial hypertension	129 (61%)	n/%, 95% CI	30/23, 17–31
Previous cardiac surgery	213 (100%)	n/%, 95% CI	19/9, 6–14
Uncomplicated dissection	129 (61%)	n/%, 95% CI	66/51, 43–60
Complicated dissection	213 (100%)	n/%, 95% CI	100/47, 40–54
Cardiac tamponade	213 (100%)	n/%, 95% CI	52/24, 19–31
Coronary malperfusion	213 (100%)	n/%, 95% CI	19/9, 6–14
Malperfusion of brachiocephalic arteries	213 (100%)	n/%, 95% CI	23/11, 7–16
Hemiplegia	213 (100%)	n/%, 95% CI	8/4, 2–7
Monoplegia	213 (100%)	n/%, 95% CI	9/4, 2–8
Paraplegia	213 (100%)	n/%, 95% CI	2/1, 0–3
Malperfusion of inner organs	213 (100%)	n/%, 95% CI	32/15, 11–20
Intestinal infarction	213 (100%)	n/%, 95% CI	0/0, 0–2
Renal malperfusion	213 (100%)	n/%, 95% CI	13/6, 4–10
Lower limb ischemia	213 (100%)	n/%, 95% CI	31/15, 10–20
Stanford type A	213 (100%)	n/%, 95% CI	207/97, 94–99
Femoral cannulation	213 (100%)	n/%, 95% CI	92/43, 37–50
Subclavian cannulation	126 (59%)	n/%, 95% CI	60/48, 39–56
Ascending aortic cannulation	111 (52%)	n/%, 95% CI	53/48, 39–57
Duration of artificial circulation (min)	204 (96%)	Me [Q1; Q3] M±SD Min–max	222 [180; 265] 225.89±64.25 60–454
Duration of aortic occlusion (min)	203 (95%)	Me [Q1; Q3] M±SD Min–max	135.0 [102.5; 170.0] 134.82±47.19 20–285
Time of circulatory arrest (min)	165 (77%)	Me [Q1; Q3] M±SD Min–max	42 [35; 61] 46.47±19.89 5–100
Aortic root replacement	213 (100%)	n/%, 95% CI	48/23, 17–29
Aortic valve replacement	213 (100%)	n/%, 95% CI	34/16, 12–21
Aortic valve plasty	213 (100%)	n/%, 95% CI	49/23, 18–29
Aortocoronary bypass surgery	213 (100%)	n/%, 95% CI	8/4, 2–7
Arch reconstruction, beveled anastomosis	213 (100%)	n/%, 95% CI	95/45, 38–51
Arch reconstruction, debranching Arch reconstruction, total arch replacement	128 (60%) 213 (100%)	n/%, 95% CI n/%, 95% CI	4/3, 1–8 47/22, 17–28
Bare-metal stent implantation	212 (99.5%)	n/%, 95% CI	55/26, 21–32
Stent-graft implantation	213 (100%)	n/%, 95% CI	37/17, 13–23
ABP	192 (90%)	n/%, 95% CI	124/65, 58–71
RBP	192 (90%)	n/%, 95% CI	68/35, 29–42
***Postoperative period***			
Without complications	211 (99%)	n/%, 95% CI	98/46, 40–53
Significant hemorrhages	211 (99%)	n/%, 95% CI	33/16, 11–21
Neurological complications (all)	207 (97%)	n/%, 95% CI	39/19, 14–25
Cerebral neurological complications	207 (97%)	n/%, 95% CI	33/16, 12–22
Spinal neurological complications	207 (97%)	n/%, 95% CI	3/1, 0–4
Myocardial infarction	209 (98%)	n/%, 95% CI	13/6, 4–10
Intestinal ischemia	207 (97%)	n/%, 95% CI	11/5, 3–9
Complete thrombosis/obliteration of the false lumen	198 (93%)	n/%, 95% CI	48/24, 19–31
Partial thrombosis/obliteration of the false lumen	195 (92%)	n/%, 95% CI	59/30, 24–37
Completely patent false lumen	195 (92%)	n/%, 95% CI	90/46, 39–53

Here: ABP is antegrade brain perfusion, RBP is retrograde brain perfusion.

The logistic regression method for single-factor and multifactor models was used for identification of lethality predictors in the remote period (Table 3). Building single-factor models of logistic regression, separate significant predictors of lethality were found. For example, coronary malperfusion and significant hemorrhages increased the probability by 3.17 and 3.22 times, respectively, however, when these covariates were included in the multifactor model, their significance reduced. Building of the multifactor model of logistic regression has demonstrated that neurological complications in the postoperative period increased the probability of lethality by 3.39 (1.24–9.18) times, and presence of the completely patent lumen by 4.17 (1.49–13.68) times.

**Table 3:** Covariate values in the logistic regression models for lethality in the remote period (n=213, of them 26 events (12.2%))

Covariates	Single-factor models		Multifactor model	
	OR (95% CI)	p	OR (95% CI)	p
Completely patent false lumen	5.35 (2.04–16.75)	0.001*	4.17 (1.49–13.68)	0.010*
Neurological complications	4.48 (1.74–11.38)	0.002*	3.39 (1.24–9.18)	0.016*
Significant hemorrhages	3.55 (1.38–8.75)	0.007*	3.22 (0.86–10.96)	0.067
Coronary malperfusion	4.02 (1.29–11.43)	0.011*	3.17 (0.73–12.01)	0.098
Malperfusion of inner organs	2.39 (0.86–6.07)	0.077		
Intestinal ischemia	3.49 (0.72–13.27)	0.082		
Partial thrombosis of the false lumen	0.33 (0.08–1.02)	0.084		
Complete thrombosis of the false lumen	0.28 (0.04–1.02)	0.097		
Without complications	0.57 (0.23–1.32)	0.201		
Body height	0.97 (0.93–1.02)	0.250		
Male gender	1.62 (0.68–4.33)	0.299		

Note: only covariates showing influence in the single-factor analysis were included in the table (p<0.3); *p<0.05.

**Figure 2.**
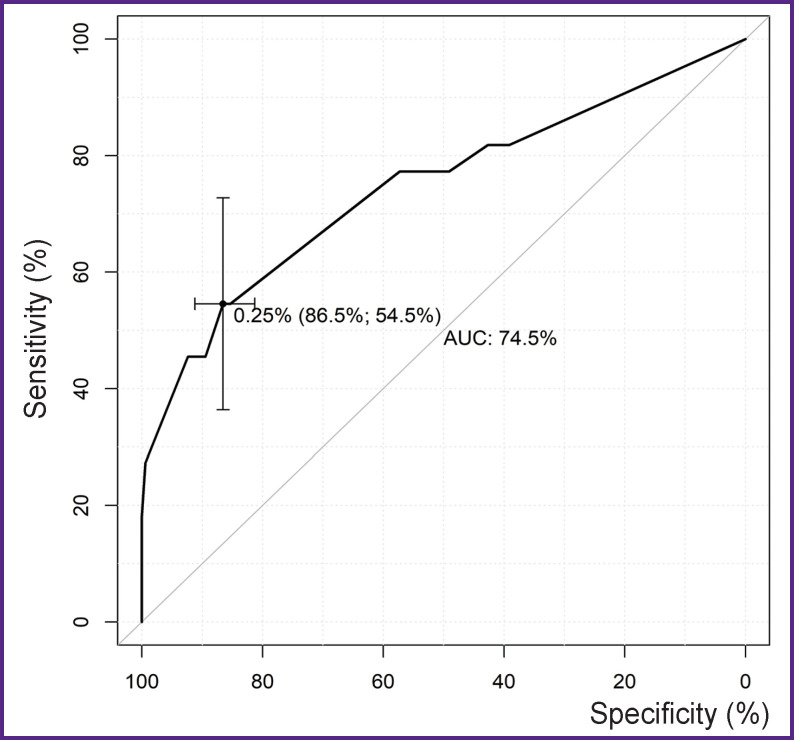
ROC curve for the multifactor model of lethality (totally over all periods; n=193)

For the threshold lethality value in the remote period equal to 24.8%, the best indicators of sensitivity (54.5%) and specificity (86.5%) in terms of the balance (Figure 2) were determined for the multifactor model using the ROC analysis.

In order to investigate prognostic properties of the multifactor model for remote lethality, a contingency table (Table 4) has been created and prognostic indicators have been calculated (Table 5). The total number of patients in the multifactor model was 193, which was 20 patients fewer than in the general sample due to the missed data in the covariates.

**Table 4:** Contingency table for the multifactor model of remote lethality (abs. number of events)

Lethality prognosis	Lethal outcome		Total
	“+”	“–”	
“+”	12	23	35
“–”	10	148	158
Total	22	171	193

**Table 5:** Prognostic indicators for the multifactor model of remote lethality

Parameters	Value (95% CI)
Frequency of method occurrences	18.1 (13.0–24.3)
Actual rate of occurrences	11.4 (7.3–16.7)
Sensitivity	54.5 (32.2–75.6)
Specificity	86.5 (80.5–91.3)

The level of significance obtained for the Hosmer– Lemeshow test (p=0.245) demonstrated goodness of fit for prognostic frequencies of the calibrated model and the actual frequency of remote lethality. The complex metrics (AUC=74.5) showed a satisfactory quality of the model classification (see Figure 2).

The logistic regression method for a single-factor and multifactor model was applied to determine predictors of aorta-related events (Table 6). Several factors had a strong influence on the occurrence of aorta-related events in the remote period. Diseases of the connective tissue increased the probability of the event by 6.68 (2.98–15.62) times, partial thrombosis of the false lumen by 2.39 (1.07–5.44) times. Aortic valve repair increased the probability of aorta-related events by 2.84 (1.13–7.17) times, which may speak of the necessity of thorough valve revision and its more frequent replacement. It should be also noted that significant bleedings in the postoperative period reduced the likelihood of aorta-related events in the remote period (0.24 (0.05–0.88) at р=0.051).

**Table 6:** Covariate values in the logistic regression models for aorta-related events in the remote follow-up period (n=213, of them 59 events (27.7%))

Covariates	Single-factor models		Multifactor model	
	OR (95% CI)	p	OR (95% CI)	p
Connective tissue diseases	5.72 (2.90–11.52)	<0.001*	6.68 (2.98–15.62)	<0.001*
Duration of aortic occlusion	1.01 (1.0–1.02)	0.015*	1.01 (1.0–1.02)	0.035*
Significant hemorrhages	0.22 (0.05–0.65)	0.015*	0.24 (0.05–0.88)	0.051
Stent-graft implantation	2.36 (1.12–4.91)	0.022*		
Hybrid prosthesis implantation	2.36 (1.12–4.91)	0.022*		
Malperfusion of brachiocephalic arteries	0.22 (0.03–0.79)	0.047*	0.26 (0.04–1.08)	0.103
Partial thrombosis of the false lumen	1.92 (0.99–3.68)	0.050	2.39 (1.07–5.44)	0.035*
Stanford type A	0.18 (0.02–0.95)	0.052		
Time of circulatory arrest (1-min increase)	0.98 (0.96–1.0)	0.057		
Complicated dissection	0.58 (0.31–1.06)	0.082		
Duration of artificial circulation (1-min increase)	1.0 (1.0–1.01)	0.101		
Aortic valve plasty	1.74 (0.87–3.42)	0.110	2.84 (1.13–7.17)	0.026*
Aortic root replacement	0.53 (0.23–1.14)	0.119		
Myocardial infarction	0.20 (0.01–1.04)	0.124		
Lower limb ischemia	0.46 (0.15–1.16)	0.127		
Ascending aorta (1-mm increase)	1.95 (0.78–5.14)	0.161		
Complete thrombosis/obliteration of the false lumen	0.58 (0.25–1.22)	0.165		
Uncomplicated aortic dissection	1.71 (0.80–3.75)	0.171		
Intestinal ischemia	0.24 (0.01–1.28)	0.176		
Subclavian cannulation	1.55 (0.72–3.37)	0.260		
Classical surgical approach	0.72 (0.39–1.31)	0.278		

Note: only covariates showing influence in the single-factor analysis were included in the table (p<0.3); * p<0.05.

**Figure 3.**
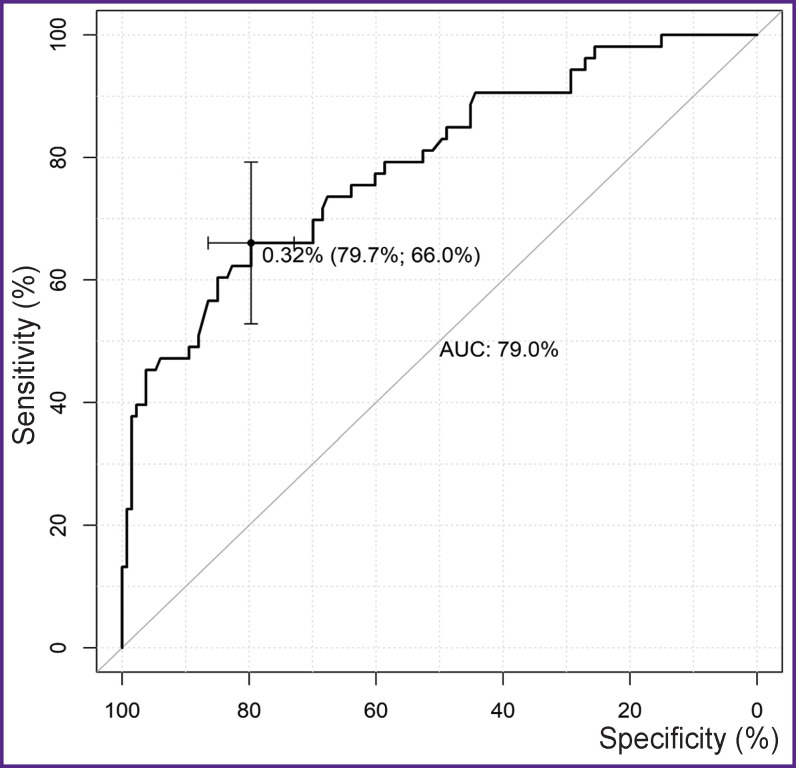
ROC curve for the multifactor model of aorta-related events (totally over all periods; n=186)

For the threshold value of the probability of aorta-related events (totally over all periods), equal to 32.4%, the best indicators of sensitivity (66%) and specificity (79.7%) (Figure 3) were determined for the multifactor model using the ROC analysis.

In order to investigate prognostic properties of the multifactor model for aorta-related events, a contingency table (Table 7) has been created and prognostic indicators have been calculated (Table 8). The total number of patients in the multifactor model was 186, being 27 patients fewer than in the general sample due to the missed data in the covariates.

**Table 7:** Contingency table for the multifactor model of aorta-related events in the remote follow-up period (abs. number of events)

Prediction of aorta-related events	Aorta-related events		Total
	“+”	“–”	
“+”	35	27	62
“–”	18	106	124
Total	53	133	186

**Table 8:** Prognostic indicators of the multifactor model of aorta-related events in the remote follow-up period

Parameters	Value (95% CI)
Frequency of method occurrences	33.3 (26.6–40.6)
Actual frequency of occurrences	28.5 (22.1–35.6)
Sensitivity	66.0 (51.7–78.5)
Specificity	79.7 (71.9–86.2)

The level of significance obtained for the Hosmer– Lemeshow (p=0.670) test demonstrated goodness of fit for prognostic frequencies of the calibrated model and the actual frequency of aorta-related events. The complex metrics (AUC=79.0%) showed a satisfactory quality of the model classification (see Figure 3).

There has been analyzed the effect of factors on the occurrence of thrombosis and complete obliteration of the aortic false lumen in 198 patients (Table 9), which was 15 patients fewer than in the general sample due to the missing data in the target indicator.

**Table 9:** Covariate values in the logistic regression models of complete thrombosis or obliteration in the group of all patients in the remote follow-up period (n=198, of them 48 events (24.2%))

Covariates	Single-factor models		Multifactor model	
	OR (95% CI)	p	OR (95% CI)	p
Hybrid prosthesis implantation	5.98 (2.78–13.16)	<0.001*	4.19 (1.90–9.44)	<0.001*
Bare-metal stent implantation	0.16 (0.04–0.47)	0.003*	0.17 (0.03–0.62)	0.021*
Hemiplegia	10.57 (2.34–73.99)	0.005*		
Beveled aggressive anastomosis	0.38 (0.18–0.76)	0.008*		
Connective tissue diseases	2.11 (1.02–4.27)	0.040*		
Body mass (1-kg increase)	0.98 (0.96–1.0)	0.053		
Absence of arterial hypertension	2.35 (0.96–5.69)	0.058		
Time of circulatory arrest	1.02 (1.0–1.04)	0.062		
Coronary malperfusion	0.18 (0.01–0.91)	0.099	0.26 (0.01–1.49)	0.216
Femoral cannulation	0.56 (0.28–1.11)	0.103		
Untreated arterial hypertension	0.49 (0.19–1.17)	0.124		
Aortic valve plasty	0.50 (0.19–1.16)	0.127		
Malperfusion of inner organs	0.43 (0.12–1.19)	0.139		
Lower limb ischemia	0.43 (0.12–1.19)	0.139		
Neurological complications	0.51 (0.18–1.22)	0.155		
Subclavian cannulation	1.77 (0.80–3.96)	0.160		
Without complications	1.55 (0.81–3.01)	0.189		
Renal malperfusion	0.27 (0.01–1.44)	0.214		
Malperfusion of brachiocephalic arteries	1.80 (0.64–4.70)	0.241		
Aortic valve replacement	0.55 (0.18–1.43)	0.256		
Marfan syndrome	1.75 (0.59–4.9)	0.292		
Male gender	0.70 (0.36–1.37)	0.294		

Note: only covariates showing influence in the single-factor analysis were included in the table (p<0.3); *p<0.05.

As one can see from Table 9, implantation of a hybrid aortic prosthesis (group 3) was the strongest predictor of false lumen thrombosis in the remote period increasing its probability by 4.19 (1.90–9.44) times. Implantation of a bare-metal stent (group 2), on the contrary, reduced false lumen thrombosis by 0.17 (0.03–0.62) times.

**Figure 4.**
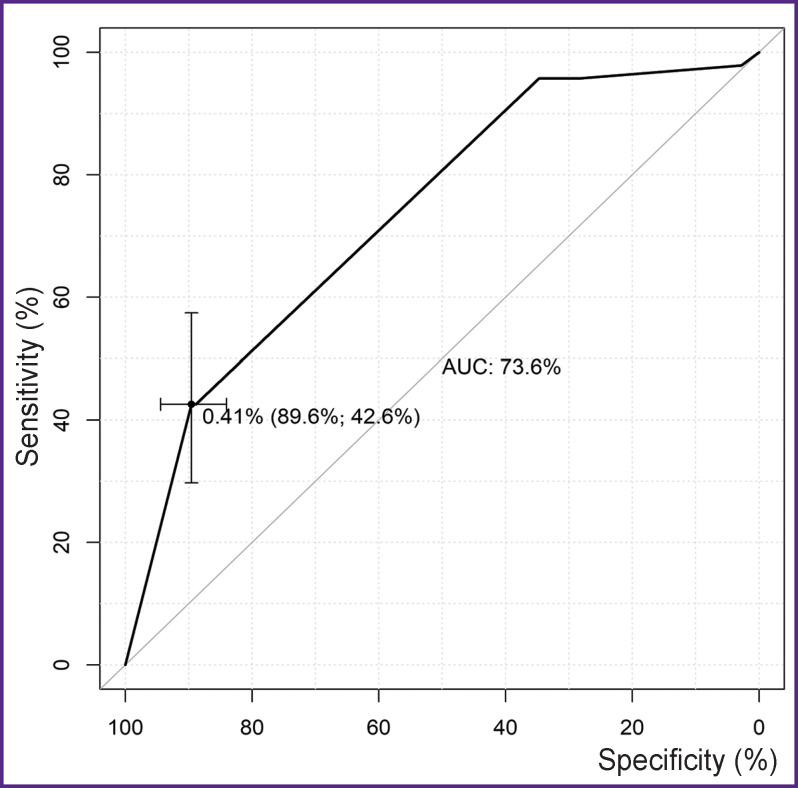
ROC curve for the multifactor model of complete thrombosis or obliteration in the remote period (n=191)

For the 40.9% threshold value of the probability of complete thrombosis/obliteration in the postoperative period, the best indicators of sensitivity (42.6%) and specificity (89.6%) (Figure 4) were determined for the multifactor model by means of the ROC analysis. Using the threshold value obtained, complete thrombosis/ obliteration of the aortic false lumen was predicted.

In order to investigate prognostic properties of the multifactor model for complete thrombosis or obliteration in the remote postoperative period, a contingency table (Table 10) has been created and prognostic indicators have been calculated (Table 11). The total number of patients in the multifactor model was 191, being 22 patients fewer than in the general sample due to the missed data in the covariates.

**Table 10:** Contingency table for the multifactor model of complete thrombosis or obliteration in the remote postoperative period (abs. number of events)

Prediction of complete thrombosis or obliteration	Complete thrombosis or obliteration		Total
	“+”	“–”	
“+”	20	15	35
“–”	27	129	156
Total	47	144	191

**Table 11:** Prognostic indicators for the multifactor model of complete thrombosis or obliteration in the remote postoperative period

Parameters	Value (95% CI)
Frequency of method occurrences	18.3 (13.1–24.6)
Actual frequency of occurrences	24.6 (18.7–31.3)
Sensitivity	42.6 (28.3–57.8)
Specificity	89.6 (83.4–94.1)

The level of significance of the Hosmer–Lemeshow (p=0.007) has shown that the prognostic values of the calibrated model do not fit the actual frequencies of complete thrombosis or obliteration in the remote postoperative period. The complex metrics (AUC=73.6) demonstrated a satisfactory quality of the model classification (see Figure 4).

To identify predictors of enlargement of the descending aortic arch diameter, the four segments of aorta (aortic arch and three descending thoracic segments) were considered as a single section. To define predictors of enlargement of the abdominal aortic arch diameter, the two aortic segments (abdominal and infrarenal) were also considered as a single section. A single-factor and then multifactor complete and optimal analysis of predictors have been carried out (Table 12).

**Table 12:** Covariate values in the logistic regression models of diameter enlargement at the level of all aortic segments in the remote follow-up period (n=213, of them 101 events (47.4%))

Covariates	Single-factor models		Multifactor model	
	OR (95% CI)	p	OR (95% CI)	p
Complete thrombosis/obliteration of the false lumen	0.31 (0.15–0.62)	0.001*	0.27 (0.12–0.59)	0.001*
Malperfusion of brachiocephalic arteries	0.27 (0.09–0.71)	0.013*	0.30 (0.08–0.97)	0.057
Complicated dissection	0.53 (0.30–0.91)	0.021*	0.59 (0.30–1.15)	0.121
Partial thrombosis/obliteration of the false lumen	2.01 (1.08–3.8)	0.029*		
Neurological complications	0.45 (0.21–0.93)	0.035*	0.30 (0.12–0.72)	0.008*
Bicuspid aortic valve	0.23 (0.03–0.94)	0.067		
Hemiplegia	0.15 (0.01–0.86)	0.078		
Stanford type A	0.17 (0.01–1.10)	0.112		
Aortic root replacement	0.59 (0.30–1.14)	0.120		
Significant hemorrhages	0.57 (0.26–1.21)	0.153		
Coronary bypass surgery	0.36 (0.05–1.59)	0.214	0.19 (0.03–0.93)	0.057
Cardiac tamponade	0.69 (0.36–1.29)	0.244		
Uncomplicated aortic dissection	1.51 (0.76–3.05)	0.244		
Beveled aggressive anastomosis	0.73 (0.42–1.26)	0.264	0.56 (0.28–1.11)	0.100

Note: only covariates showing influence in the single-factor analysis were included in the table (p<0.3); * p<0.05.

The single-factor analysis has revealed several statistically significant predictors of aorta enlargement. When building a multifactor model, complete thrombosis or obliteration of the false lumen turned out to be the strongest predictor reducing the probability of widening thoracoabdominal aorta. This predictor decreased the event probability by 0.27 (0.12–0.59) times (p=0.001). Another unexpected strong predictor reducing the probability of aorta enlargement was the development of neurological complications in the postoperative period (0.30 (0.12–0.72) at p=0.008).

**Figure 5.**
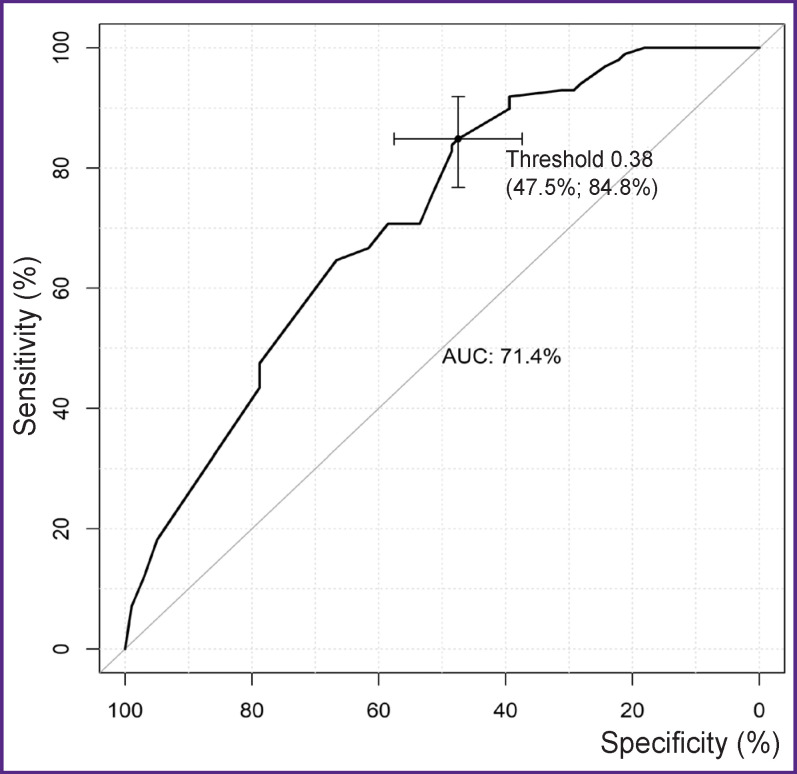
ROC curve for the multifactor model of diameter enlargement in the remote period at any level of all aortic segments (n=198)

Using the ROC analysis, the best indicators of sensitivity (84.8%) and specificity (47.5%) (Figure 5) have been determined for the multifactor model at the 38.1% threshold value for the diameter enlargement probability at the level of all aortic segments. Using the obtained threshold value, enlargement of aortic diameter at any level has been predicted.

To investigate the prognostic properties of the multifactor model of diameter enlargement at any level of all aortic segments, a contingency table (Table 13) was created and prognostic indicators were calculated (Table 14). The total number of patients in the multifactor model was 198, being 15 patients fewer than in the general sample due to the missing data in covariates.

**Table 13:** Contingency table for the multifactor model of diameter enlargement in the remote period at any level of all aortic segments (abs. number of occurrences)

Prediction of diameter enlargement at the level of all aortic segments	Diameter enlargement at the level of all aortic segments		Total
	“+”	“–”	
“+”	84	52	136
“–”	15	47	62
Total	99	99	198

**Table 14:** Prognostic indicators for the multifactor model of diameter enlargement at any level of all aortic segments in the remote follow-up period

Parameters	Values (95% CI)
Frequency of method occurrences	68.7 (61.7–75.1)
Actual frequency of occurrence	50.0 (42.8–57.2)
Sensitivity	84.8 (76.2–91.3)
Specificity	47.5 (37.3–57.8)

The level of significance obtained for the Hosmer– Lemeshow test (p=0.147) demonstrated goodness of fit for prognostic frequencies of the calibrated model and the actual frequency of diameter enlargement at the level of all segments. The complex metrics (AUC=71.4%) shows a satisfactory quality of the model classification (see Figure 5).

## Discussion

Over several recent decades, disputes on the selection of the surgical intervention volume in treating type I aortic dissection were focused on whether primary reconstruction of the proximal dissection should be done within the limits of the ascending segment and aortic arch. Evidently, hospital survival in primary intervention does not ensure the development of aorta-related events requiring surgical reinterventions since the majority of patients, who survived the primary correction of aortic dissection, have persisting dissected part of the aorta and/or distal fenestration [16].

It has been shown in some studies [4, 17] that an extended volume of the primary reconstruction of proximal dissection in the form of aortic arch repair reduces frequency of late aortic complications and improves long-term survival. At the same time, there are reports showing that complete replacement of the aortic arch does not eliminate the necessity of repeated operations or reduces remote lethality. For example, Larsen et al. [18], having analyzed the remote results of 334 patients undergone operations for complete aortic arch replacement and 907 hemiarch reconstructions, did not find any differences in a general 5-year survival (73.1 vs. 69.4%; р=0.83). In our study, the analysis of the intervention type effect on the lethality did not detect strong relations between these indicators in the remote follow-up period.

Predictors of lethality in the remote period (within 5 years) were identified using the logistic regression method. Presence of neurological complications in the postoperative period has been found to increase the probability of lethality by 3.39 (1.24–9.18) times, while completely patent lumen increased it by 4.17 (1.49–13.68). Our results agree with the data presented by Olsson et al. [19], who reported a strong impact of neurological complications on the survival of patients with aortic dissection.

Reoperations on aorta in patient survived an acute period of dissection do not happen rarely. In the study performed by Kirsch et al. [20], actuarial freedom from redo operations was 60.8±6.8% after 10 years, in the work of Fattouch et al. — 81.3% within 10 years [21]. Kim et al. [22] have shown that the rate of the reoperations did not depend on the type of aortic arch reconstruction: 5-year freedom from repeated operations was 88% in the group of the total aortic arch replacement and 92.8% in the group with the hemiarch reconstruction.

The analysis of predictors of aorta-related events after a primary reconstruction has shown that connective tissue diseases increased the probability of event occurrence by 6.68 (2.98–15.62) times. The results obtained by us were in line with the previously published data on the effect of connective tissue dysplasia on the frequency of aorta-related events in the remote period [21, 23]. Fattouch et al. [21] have detected statistically significant difference between patients with the Marfan syndrome and without it in the extent of the false lumen obliteration. According to the Cox regression analysis performed by the authors, presence of the Marfan syndrome was a predictor of a late surgical reintervention on the descending aorta.

One more important factor of the ongoing pathological remodeling of aorta and development of unfavorable outcome in the remote period is the presence of a patent false lumen [24-26]. It has been shown in some works [27, 28] that patency of the false lumen was preserved in 26.5–39.4% of cases after the first stage of reconstruction. In the process of our investigation, the presence of partial thrombosis of the false lumen was found to increase the probability of the event by 2.39 (1.07–5.44) times.

Thrombosis of the false lumen is a critically important factor influencing the lethality rate in the remote follow-up period. In the study of Tsai et al. [29], partial thrombosis of the false lumen was a key predictor of mortality (the relative risk of 2.69; 95% CI: 1.45–4.98; р=0.002). According to our results, implantation of a hybrid prosthesis was a strong predictor of complete false lumen thrombosis increasing the probability of the event by 4.19 (1.90– 9.44) times, whereas implantation of a bare-metal stent affects negatively the occurrence of thrombosis reducing its probability by 0.17 (0.03–0.62) times. This agreed with the data on a positive effect of a hybrid prosthesis implantation on remodeling of the aortic lumen [17], however, the fact of the negative influence of the bare-metal stent implantation on the remodeling has been revealed for the first time.

Since it is the presence of the patent false lumen that determines the risk of subsequent aorta dilation, one of the main criteria influencing the decision to perform intervention on aorta is enlargement of its diameter (5 cm or more) or the rate of its widening (according to the data of the sequential studies, more than 5 mm over 6 months of observation). For example, Fattori et al. [24] and Halstead et al. [30] have found that the annual rate of aorta growth was maximum in the descending segment and considerably higher in patients with an open false lumen; the mean growth rate was 1 mm/year. The initial sizes of aorta were also of significance for the outcomes of surgical aortic dissection reconstruction. It has been noted by Fattouch et al. [21] that the initial diameter of the descending aorta greater than 4.5 cm is a predictor of the late repeated intervention (hazard ratio (HR) of 5.8; 95% CI: 3.5–22.5; p=0.002).

In our study, the presence of complete thrombosis or obliteration of the false lumen appeared to be the strongest predictor diminishing the probability of widening of all aortic segments and reducing the probability of the event by 0.27 (0.12–0.59) times (p=0.001). Another unexpected strong predictor determining the probability of aortic enlargement was the development of neurological complications in the postoperative period increasing the probability of the event by 0.30 (0.12–0.72) times (p=0.008). It may be explained by the fact that the majority of neurological events in aortic dissection were connected with a more complex and lengthy intervention on the aortic arch, which in its turn increased the risk of neurological complications and the probability of thrombosis or obliteration of the false lumen. The analysis of the data for the thoracic aorta has shown that thrombosis or obliteration of the false lumen had also a significant influence on the reduction of aorta-related events in the remote period by 0.11 (0.03–0.28) times (p<0.001).

### Limitations of the study

All models calculated by us have a satisfactory quality of predicting the events of lethality, aorta-related events, false lumen thrombosis, and enlargement of aortic diameter. However, other unaccounted factors may influence the emergence of these events. For example, multifocal atherosclerosis, cardiac rhythm disorder, ischemic heart disease, oncological diseases may affect the remote lethality indicators. Therefore, further investigation of the nonlinear relations of covariates and their intersections is quite necessary to make prognostic models more exact.

## Conclusion

Our retrospective observational comparative study of the effectiveness of different types of proximal aortic reconstruction and the effect of various factors on the results has proved the negative influence of a bare-metal stent implantation on the process of postoperative aortic remodeling. At the same time, the type of intervention has no effect on the rate of aorta-related events and lethality in the remote follow-up period.
